# Supplementary material for: The genome sequence of the Pale Eggar, Trichiura crataegi (Linnaeus, 1758) (Lepidoptera: Lasiocampidae)
Source: Wellcome Open Res. 2026 Jul 15;11:447. [Version 1] doi: 10.12688/wellcomeopenres.26998.1 (PMC13562948; doi:10.12688/wellcomeopenres.26998.1)
Supplement: Peer review history

# Reviewer report: Reviewer response for version 1

**DOI:** 10.21956/wellcomeopenres.29719.r161618

**Published:** 2026-09-08

**Authors:** Jayan Duminda M Senevirathna

**Affiliations:** 1 Uva Wellassa University, Badulla, Uva Province, Sri Lanka

The reported BUSCO results indicate a relatively high level of gene-space completeness in both haplotypes; however, the authors should place these results in a broader context of assembly quality.

 More importantly, despite the comparable BUSCO completeness between the two haplotypes in the present study, only haplotype 1 has been resolved to the chromosome level, whereas haplotype 2 remains at the scaffold level. Therefore, the similar BUSCO values should not be interpreted as demonstrating equivalent overall assembly quality.

 A clear discussion of the limitations of the scaffold-level assembly of haplotype 2 is also warranted, particularly regarding its use in downstream analyses of chromosome organization, synteny, and comparative genomics.

Are sufficient details of methods and materials provided to allow replication by others?

Yes

Is the rationale for creating the dataset(s) clearly described?

Yes

Are the datasets clearly presented in a useable and accessible format?

Yes

Are the protocols appropriate and is the work technically sound?

Yes

Reviewer Expertise:

Aquatic molecular biology and biotechnology

I confirm that I have read this submission and believe that I have an appropriate level of expertise to confirm that it is of an acceptable scientific standard.

# Reviewer report: Reviewer response for version 1

**DOI:** 10.21956/wellcomeopenres.29719.r165781

**Published:** 2026-08-28

**Authors:** Rajib Deb

**Affiliations:** 1 ICAR-National Research Centre, Guwahat, Assam, India

Dataset presented here is scientifically instrumental, robust and sound.

 My minor suggestions:

 a. It would be useful to clearly state whether the reported genome size and assembly size represent the haploid genome or the combined two-haplotype assembly, to avoid ambiguity.

 b. Addition of a concise table summarizing key assembly metrics for each haplotype, including assembly size, chromosome number, N50, BUSCO completeness, QV, and k-mer completeness will be perfect

 c. The authors could briefly discuss the biological significance of identifying the Z chromosome, particularly its relevance to sex-chromosome evolution and comparative genomics in Lepidoptera.

Are sufficient details of methods and materials provided to allow replication by others?

Yes

Is the rationale for creating the dataset(s) clearly described?

Yes

Are the datasets clearly presented in a useable and accessible format?

Yes

Are the protocols appropriate and is the work technically sound?

Yes

Reviewer Expertise:

Genomics

I confirm that I have read this submission and believe that I have an appropriate level of expertise to confirm that it is of an acceptable scientific standard, however I have significant reservations, as outlined above.

# Reviewer report: Reviewer response for version 1

**DOI:** 10.21956/wellcomeopenres.29719.r161617

**Published:** 2026-07-30

**Authors:** Annabel Whibley

**Affiliations:** 1 Grapevine Improvement, Bragato Research Institute, Lincoln, New Zealand

In this Data Note, Lewis, Crowley and colleagues report the reference genome assembly of the Pale Eggar, *Trichiura crataegi*. A haplotype-resolved assembly of this relatively large insect genome is presented, with >99.8% of the haplotype assembly scaffolded into 28 pseudo-chromosomes.  The natural history section of the report is very nicely presented. Specimen identity was verified by barcoding and sequencing followed the standard pipelines for Low Input DNA extraction. I note, but this omission is not a concern, that the concentration and quality properties of the HMW extraction are not disclosed, unlike in other reports I have recently reviewed.  All protocols and software tools are carefully reported and versioned as appropriate. The quality of the assembly is supported by the standard panel of evaluation tools and all of these underscore the excellent quality of the obtained assembly. Report structure is templated, clear and comprehensive.

Are sufficient details of methods and materials provided to allow replication by others?

Yes

Is the rationale for creating the dataset(s) clearly described?

Yes

Are the datasets clearly presented in a useable and accessible format?

Yes

Are the protocols appropriate and is the work technically sound?

Yes

Reviewer Expertise:

Bioinformatics, Genomics

I confirm that I have read this submission and believe that I have an appropriate level of expertise to confirm that it is of an acceptable scientific standard.
